# Symptomatic, clinical and biomarker associations for mortality in hospitalized COVID-19 patients enriched for African Americans

**DOI:** 10.1186/s12879-022-07520-1

**Published:** 2022-06-17

**Authors:** Hassan Ashktorab, Antonio Pizuorno, Folake Adeleye, Adeyinka Laiyemo, Maryam Mehdipour Dalivand, Farshad Aduli, Zaki A. Sherif, Gholamreza Oskrochi, Kibreab Angesom, Philip Oppong-Twene, Suryanarayana Reddy Challa, Nnaemeka Okorie, Esther S. Moon, Edward Romos, Boubini Jones-Wonni, Abdoul Madjid Kone, Sheldon Rankine, Camelita Thrift, Derek Scholes, Chiamaka Ekwunazu, Abigail Banson, Brianna Mitchell, Guttu Maskalo, Jillian Ross, Julencia Curtis, Rachel Kim, Chandler Gilliard, Geetha Ahuja, Joseph Mathew, Warren Gavin, Areeba Kara, Manuel Hache-Marliere, Leonidas Palaiodimos, Vishnu R. Mani, Aleksandr Kalabin, Vijay Reddy Gayam, Pavani Reddy Garlapati, Joseph Miller, Lakshmi Gayathri Chirumamilla, Fatimah Jackson, John M. Carethers, Farin Kamangar, Hassan Brim

**Affiliations:** 1 Department of Medicine, GI Division, Cancer Center, Howard University Hospital, 2041 Georgia Avenue, N.W., Washington, DC USA; 2 Department of Pathology and Cancer Center, Department of Biochemistry & Molecular Biology, Howard University College of Medicine, Washington, DC USA; 3 College of Engineering and Technology, American University of the Middle East, Salmiya, Kuwait; 4 Division of General Internal Medicine and Geriatrics, Indiana University School of Medicine, Indianapolis, IN USA; 5 Department of Medicine, Albert Einstein College of Medicine, Bronx, NY USA; 6 Department of Trauma, Acute and Critical Care Surgery, Duke University Medical Center, Durham, NC USA; 7 Dartment of Surgery, Columbia University College of Physicians and Surgeons at Harlem Hospital, New York, NY USA; 8 Department of Medicine, Interfaith Medical Center, New York, NY USA; 9 Departments of Emergency Medicine and Internal Medicine, Henry Ford Hospital, Detroit, MI USA; 10 Division of Gastroenterology and Hepatology, Department of Internal Medicine, Department of Human Genetics and Rogel Cancer Center, University of Michigan, Ann Arbor, MI USA; 11 Department of Biology, School of Computer, Mathematical, and Natural Sciences, Morgan State University, Baltimore, MD USA

**Keywords:** COVID-19, SARS CoV-2, African American, Hispanic, Race, Ethnicity, Mortality, Hospitalized

## Abstract

**Background and Aims:**

Initial reports on US COVID-19 showed different outcomes in different races. In this study we use a diverse large cohort of hospitalized COVID-19 patients to determine predictors of mortality.

**Methods:**

We analyzed data from hospitalized COVID-19 patients (n = 5852) between March 2020- August 2020 from 8 hospitals across the US. Demographics, comorbidities, symptoms and laboratory data were collected.

**Results:**

The cohort contained 3,662 (61.7%) African Americans (AA), 286 (5%) American Latinx (LAT), 1,407 (23.9%), European Americans (EA), and 93 (1.5%) American Asians (AS). Survivors and non-survivors mean ages in years were 58 and 68 for AA, 58 and 77 for EA, 44 and 61 for LAT, and 51 and 63 for AS. Mortality rates for AA, LAT, EA and AS were 14.8, 7.3, 16.3 and 2.2%. Mortality increased among patients with the following characteristics: age, male gender, New York region, cardiac disease, COPD, diabetes mellitus, hypertension, history of cancer, immunosuppression, elevated lymphocytes, CRP, ferritin, D-Dimer, creatinine, troponin, and procalcitonin. Use of mechanical ventilation (p = 0.001), shortness of breath (SOB) (p < 0.01), fatigue (p = 0.04), diarrhea (p = 0.02), and increased AST (p < 0.01), significantly correlated with death in multivariate analysis. Male sex and EA and AA race/ethnicity had higher frequency of death. Diarrhea was among the most common GI symptom amongst AAs (6.8%). When adjusting for comorbidities, significant variables among the demographics of study population were age (over 45 years old), male sex, EA, and patients hospitalized in New York. When adjusting for disease severity, significant variables were age over 65 years old, male sex, EA as well as having SOB, elevated CRP and D-dimer. Glucocorticoid usage was associated with an increased risk of COVID-19 death in our cohort.

**Conclusion:**

Among this large cohort of hospitalized COVID-19 patients enriched for African Americans, our study findings may reflect the extent of systemic organ involvement by SARS-CoV-2 and subsequent progression to multi-system organ failure. High mortality in AA in comparison with LAT is likely related to high frequency of comorbidities and older age among AA. Glucocorticoids should be used carefully considering the poor outcomes associated with it. Special focus in treating patients with elevated liver enzymes and other inflammatory biomarkers such as CRP, troponin, ferritin, procalcitonin, and D-dimer are required to prevent poor outcomes.

## Introduction

By April, 2022, the SARS-CoV-2 virus that causes COVID-19 had infected 79 million Americans and led to over 976,516 deaths[1]. The United States is one of the most ethnically and racially diverse countries in the world encompassing 13.4% African Americans (AA) and 18.5% Latinos/Latinas (LAT) [1]. Disparities among various ethnic groups span many aspects of health [2–4], and specific racial/ethnic minority populations have been disproportionately affected by the morbidity and mortality related to COVID-19 [5–7]. Reports across the US indicate that African American and Hispanic populations have higher COVID-19 related hospitalization and mortality than Caucasians. Nationwide, AA have died at 1.4 times the rate of white people [8, 9]. The presence of racial and ethnic disparities in COVID-19 morbidity and mortality is partly due to abiding economic and educational disadvantages that have culminated in decreased healthcare utilization and prevalent distrust of the healthcare system among minority individuals [10].

A contributory cause for such a disproportionate impact of COVID-19 in AA and LAT racial/ethnic minority populations may be the higher prevalence of chronic conditions or comorbidities when compared to European Americans (EA) [6, 11, 12]. Members of these ethnic/racial groups may disproportionately hold occupations that do not permit adequate physical distancing, increasing the potential for environmental infection and transmission is enhanced [13]. The majority of the AA and LAT are essential workers/ frontline workers who need to work in person and are at even higher risk of contracting COVID-19. There may also be differences in the provisions of hospital care by racial/ethnic groups [4, 12], but there has not been adequate data on hospital outcomes by race or ethnicity that adjusts for potential confounders. Four major reasons may explain the higher risk for COVID-19, Age, co-morbidities (such as diabetes, obesity, hypertension, cardiovascular Asthma, chronic obstructive pulmonary disease (COPD), and chronic kidney disease), access to health care and social economic status particularly for minority[4, 12, 14]. The race and ethnicities to be strong risk factors for infection and COVID-19 includes those of African descent and Native Americans[12, 14].

In this study, we examined a hospitalized SARS-CoV-2-positive cohort data enriched for AA including gastrointestinal symptoms, clinical markers, and comorbidities from 8 hospitals nationwide to determine COVID-19 mortality predictors.

## Materials and methods

### Patient selection

In this retrospective study we collected de-identified data and reviewed anthropometric, clinical, serum laboratory tests and outcomes data of 5852 COVID-19 patients hospitalized at eight institutions in the United States between March 2020- August 2020. This study was approved by Howard University Institutional Review Board (IRB) and the respective IRBs of all participating centers [except data from Albert Einstein College of Medicine, Bronx, NY where data was accessed [15] after clarifications provided by the authors. An excel file template was shared with our collaborators to streamline and homogenize the process of data collection and database construction. The total number of patients included in this study was 5,852. However, the total number for each variable in overall analysis varies due to the missing values.

### Inclusion and exclusion criteria

The following inclusion criteria were used to select patients and studies that were involved in our analysis: confirmed diagnosis of SARS-CoV-2 by PCR and hospitalized for COVID-19, without restrictions based on number of patients, and without distinction on sex, age, treatment, manifestations, comorbidities, or outcomes. Exclusion criteria included: studies where the cohort did not include AA or LAT patients, studies from outside of the US, studies where the cases were not confirmed by PCR, studies with incomplete symptoms and comorbidities, and those with overlapping data.

### Selected studies

De-identified data was obtained from 8 centers: Phoebe Putney Health System in Southwest Georgia (SWG) from March 2-May 6, 2020), Interfaith Medical Center, Brooklyn (NYK) from March 1-April 9, 2020, Harlem Hospital Columbia University (NYM) during March and April 2020, Providence Community Health Centers (RIH) from March 19-April 29, 2020, Montefiore Medical Center in Bronx (NYB) from March 1-April 12, 2020; Large academic institutions served as a referral center and enrolled patients into local COVID-19 databases, including for the state of Indiana (Indiana University School of Medicine, IUM) from March 1-March 31, 2020, Henry Ford Hospital Detroit (HFD) from March 7-April 30, 2020**,** and Howard University Hospital (HUH) from February 28-August 9, 2020.

### Database creation and data homogenization

From the selected studies, we obtained individually de-identified data. We created tables containing the following information: study author (location, hospital or city and state), date of the report, location, confirmed cases, deaths, age, symptoms (fever, chest pain, cough, shortness of breath, abdominal pain, anorexia, diarrhea, nausea, vomiting, fatigue, headache), comorbidities (cardiac disease, coronary artery disease, asthma, diabetes mellitus, hypertension, immunocompromised status, cancer and alcohol intake), laboratory clinical values (sodium, potassium, blood urea nitrogen (BUN), creatinine, hemoglobin, lymphocyte count, CPK, C-Reactive Protein (CRP), D-dimer, troponin, ferritin, liver function enzymes, and procalcitonin), and treatment (Hydroxychloroquine, Glucocorticoid, Intubation/Mechanical ventilation and outcome (alive or dead). For laboratory clinical values, reference laboratory values were used to determine normal from abnormal values (Low and High values), such as for lymphopenia and lymphocytosis. All data were coded to streamline for the statistical analysis. The collected data were checked, cross validated for data completeness and accuracy and amended in unlikely cases of error, by contacting the data collectors and revisiting patient charts.

### Statistical analysis

Patient demographics, symptoms, underlying comorbidities, treatment, and outcomes were compared among AA, EA, LAT, and other ethnic groups. Predictors of hospital mortality were evaluated by using logistic and/or multiple logistic regression using four models to assess the effect of each risk factor: OR1: no adjustment; OR2: adjusted for gender, age, ethnicity, and center; OR3: the OR2 model further adjusted for comorbidities; and OR4: the OR3 model further adjusted for disease severity. Center was treated as independent categorical variable with 8 categories and fitted to the logistic regression model as a categorical variable. Regression models were controlled for disease severity by including the indicators of disease severity such as shortness of breath, CRP, and D-dimer in the logistic regression model as independent variables. In each analysis, odds ratios (ORs) and associated 95% confidence intervals were calculated. The 95% confidence interval was investigated to see if it contains unity. Confidence intervals that included one were considered not statistically significant.

## Results

### Overall and race-specific mortality

Our study collected data from 5,858 patients from 8 different hospitals/centers across the United States of whom 5,448 had data available on race/ethnicity (Table 1). Of the total cohort, 3,662 (62.6%) patients were AA, 1,407 (24%) were EA, 286 (5%) were LAT, and 93 (1.6%) were Asian Americans (AS). The overall mortality rate was 14.5% (795 deaths) for all groups combined. The mortality rates varied by racial group: 14.8% for AA, 16.3% for EA, 7.3% for LAT and 2.2% for AS. There were substantial differences in age and sex distribution across these racial groups. The median age (in years) was 61 for AA, 62 for EA, 43 for LAT, and 51 for AS. After adjusting for age, sex, and center, the OR (95% CI) for mortality, compared to AA, was 1.35 (1.11–1.64) for EA, 0.97 (0.56–1.66) for LAT, and 0.25 (0.06–1.04) for AS (Table 2). After adjusting for comorbidities and disease severity, the differences remained.

**Table 1:** Demographics, Clinical Manifestations and Comorbidities of COVID-19 patients

	African-Americans	European-Americans	Latin-Americans	Asian-Americans
Discharge status, demographic and anthropometric indices				
Discharge status (n = 5448)	3662	1407	286	93
Alive (n = 4653)	3120 (85.2)	1177 (83.7)	265 (92.7)	91 (97.8)
Dead (n = 795)	542 (14.8)	230 (16.3)	21 (7.3)	2 (2.2)
Sex (n = 5449)				
Female (n = 2892)	1936 (52.9)	735 (52.2)	119 (41.5)	40 (43)
Male (n = 2557)	1726 (47.1)	672 (47.8)	168 (58.5)	53 (57)
Age (n = 5449)	3663	1407	287	93
(Median, interquartile range)	(61, 49, 71)	(62, 48, 78)	(43, 30 58)	(51, 40.5, 63)
BMI (n = 5074)				
Normal (n = 1174)	699 (19.8)	388 (40.3)	60 (40.3)	27 (33.8)
Overweight (n = 1321)	863 (24.4)	378 (28.9)	48 (32.2)	32 (40)
Obese (n = 2579)	1976 (55.9)	541 (41.4)	41 (27.5)	21 (26.3)
Admitting center (n = 5450)				
Michigan-Detroit	2040 (55.7)	1172 (83.3)	14 (4.9)	74 (79.6)
NY-New York	466 (12.7)	0 (0)	0 (0)	0 (0)
Indiana	90 (2.5)	37 (2.6)	7 (2.4)	5 (5.4)
Rhode Island	0 (0)	0 (0)	105 (36.6)	0 (0)
NY-Bronx	102 (2.8)	69 (4.9)	0 (0)	0 (0)
NY-Manhattan	114 (3.1)	1 (0.1)	58 (20.2)	11 (11.8)
DC-Howard	257 (7)	26 (1.8)	103 (35.9)	1 (1.1)
Georgia	594 (16.2)	102 (7.2)	0 (0)	2 (2.2)
Baseline comorbidities				
Cardiac disease (n = 4142)				
No (n = 3596)	2379 (86.2)	1040 (86.9)	105 (95.5)	72 (96)
Yes (n = 546)	381 (13.8)	157 (13.1)	5 (4.5)	3 (4)
COPD (n = 4597)				
No (n = 4144)	2673 (90.9)	1208 (87.5)	173 (94)	90 (97.8)
Yes (n = 453)	267 (9.1)	173 (12.5)	11 (6)	2 (2.2)
Asthma (n = 4353)				
No (n = 3923)	2534 (88.9)	1240 (92.3)	67 (93.1)	82 (94.3)
Yes (n = 430)	316 (11.1)	104 (7.7)	5 (6.9)	5 (5.7)
Diabetes mellitus (n = 5440)				
No (n = 3583)	2234 (61)	1060 (75.4)	217 (77.2)	72 (77.4)
Yes (n = 1857)	1426 (39)	346 (24.6)	64 (22.8)	21 (22.6)
Hypertension (n = 5437)				
No (n = 2296)	1279 (35)	762 (54.2)	194 (69.3)	61 (65.6)
Yes (n = 3141)	2379 (65)	644 (45.8)	86 (30.7)	32 (34.4)
History of cancer (n = 4308)				
No (n = 3945)	2610 (92.4)	1235 (89.5)	20 (95.2)	80 (98.8)
Yes (n = 363)	216 (7.6)	145 (10.5)	1 (4.8)	1 (1.2)
Immune suppression (n = 1948)				
No (n = 1843)	1420 (94.4)	214 (92.2)	201 (98.5)	8 (100)
Yes (n = 105)	84 (5.6)	18 (7.8)	3 (1.5)	0 (0)
Presenting symptoms				
Fever (n = 5443)				
No (n = 3811)	2471 (67.5)	1141 (81.2)	130 (45.6)	69 (74.2)
Yes (n = 1632)	1188 (32.5)	265 (18.8)	155 (54.4)	24 (25.8)
Headache (n = 4274)				
No (n = 4109)	2662 (97.3)	1317 (98.1)	56 (47.1)	74 (97.4)
Yes (n = 165)	74 (2.7)	26 (1.9)	63 (52.9)	2 (2.6)
Cough (n = 5260)				
No (n = 3709)	2395 (67.6)	1153 (82)	94 (41.4)	67 (81.7)
Yes (n = 1551)	1150 (32.4)	253 (18)	133 (58.6)	15 (18.3)
Shortness of breath (n = 5156)				
No (n = 3147)	2056 (57.9)	966 (68.8)	62 (51.2)	63 (76.8)
Yes (n = 2009)	1492 (42.1)	439 (31.2)	59 (48.8)	19 (23.2)
Chest pain (n = 4308)				
No (n = 4125)	2704 (95.7)	1321 (95.7)	19 (90.5)	81 (100)
Yes (n = 183)	122 (4.3)	59 (4.3)	2 (9.5)	0 (0)
Fatigue (n = 4696)				
No (n = 4281)	2905 (89.6)	1234 (95.4)	68 (80)	74 (97.4)
Yes (n = 415)	336 (10.4)	60 (4.6)	17 (20)	2 (2.6)
Nausea (n = 4966)				
No (n = 4694)	3202 (93.4)	1303 (97.7)	108 (90)	81 (98.8)
Yes (n = 272)	228 (6.6)	31 (2.3)	12 (10)	1 (1.2)
Vomiting (n = 4978)				
No (n = 4798)	3293 (95.8)	1312 (98.2)	113 (91.9)	80 (97.6)
Yes (n = 180)	144 (4.2)	24 (1.8)	10 (8.1)	2 (2.4)
Abdominal pain (n = 5130)				
No (n = 4950)	3402 (96.2)	1311 (98.3)	145 (86.8)	92 (98.9)
Yes (n = 180)	135 (3.8)	22 (1.7)	22 (13.2)	1 (1.1)
Diarrhea (n = 4637)				
No (n = 4322)	2833 (92.6)	1251 (95.9)	152 (84)	86 (94.5)
Yes (n = 315)	228 (7.4)	53 (4.1)	29 (16)	5 (5.5)
Anorexia (n = 5113)				
No (n = 4853)	3322 (94.4)	1307 (98.1)	137 (80.6)	87 (93.5)
Yes (n = 260)	196 (5.6)	25 (1.9)	33 (19.4)	6 (6.5)
Laboratory results (Median, interquartile range)				
Hemoglobin (n = 2903)	1872	967	20	44
Normal (n = 1158, 39.9)	712 (38)	415 (42.9)	13 (65)	18 (40.9)
Low (n = 1712, 59)	1135 (60.6)	544 (56.3)	7 (35)	26 (59.1)
Elevated (n = 33, 1.1)	25 (1.3)	8 (0.8)	0 (0)	0 (0)
Lymphocyte count (n = 3965)				
Normal (n = 1627, 41)	1187 (43.8)	354 (32.5)	67 (56.3)	19 (41.3)
Low (n = 2276, 57.4)	1480 (54.6)	724 (66.5)	45 (37.8)	27 (58.7)
Elevated (n = 62, 1.6)	44 (1.6)	11 (1)	7 (5.9)	0 (0)
CPK (n = 1897)				
Normal (n = 982, 51.8)	499 (42.8)	455 (65.8)	10 (90.9)	18 (62.1)
Elevated (n = 915, 48.2)	666 (57.2)	237 (34.2)	1 (9.1)	11 (37.9)
CRP (n = 2980)				
Normal (n = 187, 6.3)	132 (6.6)	30 (3.6)	23 (21.3)	2 (5.7)
Elevated (n = 2793, 93.7)	1880 (93.4)	795 (96.4)	85 (78.7)	33 (94.3)
D-Dimer (n = 2679)				
Normal (n = 449, 16.8)	275 (15.6)	146 (18.6)	20 (20)	8 (22.2)
Elevated (n = 2230, 83.2)	1484 (84.4)	638 (81.4)	80 (80)	28 (77.8)
Troponin (n = 2867)				
Normal (n = 1845, 64.4)	1197 (63.5)	582 (64.8)	35 (81.4)	31 (75.6)
Elevated (n = 1022, 35.6)	688 (36.5)	316 (35.2)	8 (18.6)	10 (24.4)
Procalcitonin (n = 2941)				
Normal (n = 1408, 47.9)	879 (43.8)	466 (59.4)	46 (40)	17 (53.1)
Elevated (n = 1533, 52.1)	1130 (56.2)	319 (40.6)	69 (60)	15 (46.9)
Ferritin (n = 2914)				
Normal (n = 862, 29.6)	511 (25.9)	314 (39)	28 (28.6)	9 (24.3)
Low (n = 31, 1.1)	19 (1)	11 (11.4)	0 (0)	1 (2.7)
Elevated (n = 2021, 69.4)	1443 (73.1)	481 (59.7)	70 (71.4)	27 (73)
LDH (n = 726)				
Normal (n = 154, 21.2)	113 (19.9)	40 (33.6)	0 (0)	1 (16.7)
Elevated (n = 572, 78.8)	454 (80.1)	79 (66.4)	34 (100)	5 (83.3)
AST (n = 3356)				
Normal (n = 1498 (44.6)	996 (44.3)	433 (46.9)	55 (39)	14 (31.8)
Elevated (n = 1858, 55.4)	1251 (55.7)	491 (53.1)	86 (61)	30 (68.2)
ALT (n = 2760)				
Normal (n = 2412, 87.4)	1533 (88.4)	767 (90.7)	81 (58.3)	31 (75.6)
Elevated (n = 348, 12.6)	201 (11.6)	79 (9.3)	58 (41.7)	10 (24.4)
Interventions				
Glucocorticoids (n = 3419)				
No (n = 1703)	1249 (51.3)	364 (42.3)	82 (81.2)	8 (33.3)
Yes (n = 1716)	1184 (48.7)	497 (57.7)	19 (18.8)	16 (66.7)
Mechanical Ventilation (n = 4944)				
No (n = 4262)	2816 (84.1)	1162 (89.5)	212 (96.8)	72 (93.5)
Yes (n = 682)	533 (15.9)	137 (10.5)	7 (3.2)	5 (6.5)

*The total number for each variable in overall columns may not be the same due to missing/not collected values. *CAD* coronary artery disease

**Table 2:** Predictors of mortality and Odds Ratios for mortality were generated on raw data (OR1), after adjustment for Gender/Age/Center/Ethnicity (OR2), after adjustment for Comorbidities (OR3) and after adjustment for disease severity (Shortness of breath/CRP/D-dimer, OR4)

	Alive N (%)	Dead N (%)	OR 1 (95% CI)	OR 2 (95% CI)	OR 3 (95% CI)	OR 4 (95% CI)
Age (n = 5852)						
< 35 (n = 652)	631 (12.6)	21 (2.5)	–	–	–	–
35 to 44 (n = 612)	581 (11.6)	31 (3.7)	1.60 (0.91–2.82)	1.38	1.26 (0.71–2.25)	0.60 (0.23–1.55)
45 to 54 (n = 937)	878(17.5)	59 (7.1)	2.02 (1,21–3.36)	1.64	1.40 (0.82–2.36)	0.84 (0.37–1.88)
55 to 64 (n = 1264)	1145 (22.8)	119 (14.3)	3.12 (1.94–5.02)	2.34	1.84 (1.13–3.01)	0.87 (0.40–1.86)
65 to 74 (n = 1169)	951 (18.9)	218 (26.2)	6.89 (4.35–10.90)	5.03	3.77 (2.34–6.08)	2.39 (1.14–4.99)
≥ 75 (n = 1218)	835 (16.6)	383 (46.1)	13.78 (8.78–21.64)	10.13	7.43 (4.64–11.89)	4.24 (2.04–8.80)
Sex (n = 5852)						
Female (n = 3087)	2726 (54.3)	361 (43.4)	–	–	–	–
Male (n = 2765)	2295 (45.7)	470 (56.6)	1.55 (1.33 – 1.79)	1.48 (1.26–1.74)	1.49 (1.27–1.76)	1.47 (1.17–1.84)
Admitting center (n = 5853)						
Detroit (n = 3674)	3267 (65.1)	407 (49)				
New York (n = 468)	316 (6.3)	152 (18.3)	1.56 (1.24–1.95)	3.78	4.02 (3.13–5.18)	
Indiana (n = 140)	118 (2.3)	22 (2.6)	3.86 (3.10–4.80)	1.48	1.44 (0.88–2.36)	
Rhode Island (n = 105)	105 (2.1)	0 (0)	1.49 (0.93–2.38)	.000	00	
Bronx (n = 171)	133 (2.6)	38 (4.6)	0.00 (0.00–0.00)	2.25	2.14 (1.44–3.19)	
Manhattan (n = 184)	152 (3)	32 (3.9)	2.29 (1.57–3.33)	1.54	1.59 (1.01–2.50)	1.32 (0.62–2.82)
Howard (n = 399)	335 (6.7)	64 (7.7)	1.69 (1.13–2.50)	1.93	1.88 (1.35–2.62)	1.53 (1.01–2.31)
Georgia (n = 712)	596 (11.9)	116 (14)	1.53 (1.15–2.04)	1.55	1.52 (1.19–1.94)	1.12 (0.81–1.56)
Ethnicity (n = 5448)						
African American (n = 3662)			–	–	–	–
European American (n = 1407)	3120 (67.1)	542 (68.2)	1.12 (0.95–1.33)	1.35	1.45 (1.19–1.77)	1.47 (1.15–1.88)
Latin (n = 286)	1177 (25.3)	230 (28.9)	0.45 (0.29–0.71)	.97	1.06 (0.61–1.83)	0.82 (0.33–2.02)
Asian (n = 93)	265 (5.7)	21 (2.6)	0.12 (0.3–0.51)	.25	0.27 (0.06–1.13)	00
BMI (n = 5073)						
Normal (n = 1174)	946 (22)	228 (29.3)	–	–		
Overweight (n = 1320)	1101 (25.6)	219 (28.2)	0.82 (0.67–1.01)	.93		
Obese (n = 2579)	2249 (52.4)	330 (42.5)	0.60 (0.50–0.73)	1.02		
Baseline comorbidities						
Cardiac Disease (n = 4141)						
No (n = 3595)	3148 (88.6)	447 (76)	–	–		
Yes (n = 546)	405 (11.4)	141 (24)	2.45 (197–3.04)	1.55 (1.22–1.96)		
COPD (n = 4597)						
No (n = 4144)	3675 (91.5)	469 (80.9)	–	–		
Yes (n = 453)	342 (8.5)	111 (19.1)	2.54 (2.01–3.21)	1.62 (1.25–2.08)		
Asthma (n = 4353)						
No (n = 3923)	3412 (89.9)	511 (91.6)	–	–		
Yes (n = 430)	383 (10.1)	47 (8.4)	0.81 (0.59–1.12)	1.01 (0.72–1.42)		
Diabetes Mellitus (n = 5436)						
No (n = 2295)	2073 (44.7)	222 (28)	–	–		
Yes (n = 3141)	2569 (55.3)	572 (72)	1.83 (1.57–2.14)	1.59 (1.26–1.75)		
Hypertension (n = 4308)						
No (n = 3945)	3469 (92.3)	476 (86.9)	–	–		
Yes (n = 363)	291 (7.7)	72 (13.1)	2.07 (1.76–2.45)	1.26 (1.05–1.53)		
History of cancer (n = 4308)						
No (n = 3945)	3469 (92.3)	476 (86.9)	–	–		
Yes (n = 363)	291 (7.7)	72 (13.1)	1.73 (1.12–2.68)	0.97 (0.72–1.30)		
Immune suppression (n = 1947)						
No (n = 1842)	1484 (95.3)	358 (92)	–	–		
Yes (n = 105)	74 (4.7)	31 (8)	1.73 (1.12–2.68)	1.7 (1.12–2.79)		
Presenting symptoms						
Fever (n = 5442)						
No (n = 3810)	3269 (70.3)	541 (68.1)	–	–		
Yes (n = 1632)	1379 (29.7)	253 (31.9)	1.10 (0.94–1.30)	0.84 (0.69–1.02)		
Headache (n = 4274)						
No (n = 4109)	3859 (95.8)	520 (98.9)	–	–		
Yes (n = 165)	159 (4.2)	6 (1.1)	0.26 (0.11–0.59)	0.49 (0.20–1.21)		
Cough (n = 5259)						
No (n = 3708)	3188 (70.9)	520 (68.2)	–	–		
Yes (n = 1551)	1309 (29.1)	242 (31.8)	1.13 (0.96–1.33)	0.89 (0.72–1.10)		
Shortness of breath (n = 5155)						
No (n = 3146)	2811 (64)	335 (43.9)	–	–		
Yes (n = 2009)	1581 (36)	428 (56.1)	2.27 (1.94–2.65)	1.91 (1.61–2.28)	1.81 (1.43–2.28)	1.92 (1.53–2.40)
Chest pain (n = 4308)						
No (n = 4125)	3594 (95.6)	531 (96.9)	–	–		
Yes (n = 183)	166 (4.4)	17 (3.1)	0.69 (0.41–1.15)	0.81 (0.47–1.40)		
Fatigue (n = 4695)						
No (n = 4280)	3691 (91.6)	589 (88.3)	–	–		
Yes (n = 415)	337 (8.4)	78 (11.7)	1.45 (1.11–1.88)	0.68 (0.50–0.93)		
Nausea (n = 4965)						
No (n = 4693)	4018 (94.6)	675 (94)	–	–		
Yes (n = 272)	229 (5.4)	43 (6)	1.11 (0.79–1.56)	0.74 (0.51–1.08)		
Vomiting (n = 4977)						
No (n = 4797)	4095 (96.2)	702 (97.5)	–	–		
Yes (n = 180)	162 (3.8)	18 (2.5)	0.64 (0.39–1.06)	0.65 (0.39–1.11)		
Abdominal pain (n = 5129)						
No (n = 4949)	4222 (96.3)	727 (97.7)	–	–		
Yes (n = 180)	163 (3.7)	17 (2.3)	0.60 (0.36–1.00)	0.58 (0.34–0.99)		
Diarrhea (n = 4636)						
No (n = 4321)	3707 (93.6)	614 (90.7)	–	–		
Yes (n = 315)	252 (6.4)	63 (9.3)	1.50 (1.13–2.01)	1.05 (0.75–1.46)		
Anorexia (n = 5112)						
No (n = 4852)	4154 (95.1)	698 (94.1)	–	–		
Yes (n = 260)	216 (4.9)	44 (5.9)	1.21 (0.86–1.69)	0.92 (0.63–1.35)		
Laboratory Results (quartiles determined based on the entire study population;						
Hemoglobin (n = 2903)						
Normal (n = 1158)	1010 (40.8)	148 (34.8)	–	–		
Low (n = 1712)	1441 (58.2)	271 (63.8)	1.28 (1.03–1.59)	1.23 (0.97–1.56)		
Elevated (n = 33)	27 (1.1)	6 (1.4)	1.52 (0.62 – 3.74)	1.61 (0.62–4.18)		
Lymphocyte count (n = 3965)						
Normal (n = 1627)	1463 (43.5)	164 (27.3)	–	–		
Low (n = 2276)	1857 (55.2)	419 (69.8)	2.01 (1.65–2.44)	1.63 (1.32–2.00)		
Elevated (n = 62)	45 (1.3)	17 (2.8)	3.37 (1.88–6.02)	2.53 (1.35–4.75)		
CPK (n = 1897)						
Normal (n = 982)	826 (53.1)	156 (45.9)	–	–		
Elevated (n = 915)	731 (46.9)	184 (54.1)	1.33 (1.05–1.68)	1.60 (1.23–2.09)		
CRP (n = 2980)						
Normal (n = 187)	176 (7.2)	11 (2.1)	–	–	–	–
Elevated (n = 2793)	2279 (92.8)	514 (97.9)	3.60 (1.94–6.68)	4.00 (2.06–7.75)	3.39 (1.60–7.17)	2.78 (1.35–5.73)
D-Dimer (n = 2679)						
Normal (n = 449)	433 (19.7)	16 (3.3)	–	–	–	–
Elevated (n = 2230)	1765 (80.3)	465 (96.7)	7.31 (4.28–11.8)	4.75 (2.82–8.02)	4.15 (2.44–7.05)	4.25 (2.50–7.21)
Troponin (n = 2867)						
Normal (n = 1845)	1686 (70.9)	159 (32.6)	–	–		
Elevated (n = 1022)	693 (29.1)	329 (67.4)	5.03 (4.08–6.20)	4.12 (3.26–5.22)		
Procalcitonin (n = 2491)						
Normal (n = 1408)	1266 (62.9)	142 (25.8)	–	–		
Elevated (n = 1533)	1125 (47.1)	408 (74.2)	3.23 (2.62–3.97)	3.86 (3.04–4.90)		
Ferritin (n = 2914)						
Normal (n = 862)	769 (32.1)	93 (17.9)	–	–		
Elevated (n = 2021)	1598 (66.8)	423 (81.2)	2.18 (1.72–2.78)	2.32 (1.79–3.02)		
Low (n = 31)	26 (1.1)	5 (1)	1.59 (0.59–4.24)	2.17 (0.76–6.22)		
LDH (n = 726)						
Normal (n = 154)	141 (23.7)	13 (10)	–	–		
Elevated (n = 572)	455 (76.3)	117 (90)	2.78 (1.52–5.09)	3.36 (1.77–6.37)		
AST (n = 3356)						
Normal (n = 1498)	1335 (48.4)	163 (27.2)	–	–		
Elevated (n = 1858)	1421 (51.6)	437 (72.8)	2.51 (2.07–3.06)	2.63 (2.13–3.24)		
ALT (n = 2760)						
Normal (n = 2412)	2004 (87.9)	163 (27.2)				
Elevated (n = 348)	277 (12.1)	437 (72.8)				
Interventions						
Glucocorticoids (n = 3418)						
No (n = 1702)	1424 (52.9)	278 (38.4)	–	–		
Yes (n = 1716)	1270 (47.1)	446 (61.6)	1.79 (1.52–2.12)	1.96 (1.61–2.39)		
Mechanical Ventilation (n = 4943)						
No (n = 4261)	3942 (92.8)	319 (45.8)	–	–		
Yes (n = 682)	305 (7.2)	377 (54.2)	15.2 (12.6–18.4)	15.44 (12.45–19.15)		

### Hypertension was the most frequent comorbidity among all races

The frequency and percentages for comorbidities, presenting symptoms, laboratory findings, and treatments for all patients were analyzed by race (Table 1). The most common comorbidities were hypertension (57.7%), diabetes mellitus (34.1%), cardiac disease (13.1%), COPD (9.8%), asthma (9.8%), history of cancer (8.4%) and immunosuppression (5.3%). Hypertension was the most common comorbidity in all races (65% in AA, 45.8% in EA, 30.7% in LAT and 34.4% in AS) followed by diabetes mellitus (39% in AA, 24.6% in EA, 22.8% in LAT and 22.6 in AS). Overall, AA had the highest prevalence of comorbidities over other races/ethnicities.

### Shortness of Breath (SOB) was the most frequent presenting symptoms among all races

The most common presenting symptoms were shortness of breath (38.9%), cough (29.4%%), and fever (29.9%). The main GI symptoms were diarrhea (6.7%), nausea (5.4%), vomiting (3.6%), and abdominal pain (3.5%). Overall diarrhea was the most common GI symptom among all the races (7.4% in AA, 4.1% in EA, 16% in LAT and 5.5% in AS) followed by nausea (6.6% in AA, 2.3% in EA, 10% in LAT and 1.2% in AS) except for LAT where the second most common symptom was abdominal pain (13.2%) and AS for whom vomiting was second (2.4%). In aggregate, LAT patients had the highest prevalence of symptoms over other races/ethnicities.

### Inflammatory serum markers were commonly elevated among all races

Measurements of systemic inflammation, such as CRP, D-dimer, procalcitonin, and ferritin were elevated in the majority of hospitalized COVID-19 patients irrespective of race/ethnicity (Table 1). Other commonly affected laboratory values included: presence of anemia and lymphocytopenia, elevated CPK and troponin, elevated LDH, and elevated AST but not ALT. Among races/ethnicities, AA had the highest prevalence of increased serum inflammatory markers.

### Predictors of mortality

Age was a very strong predictor of mortality from COVID-19 (Table 2). Compared to individuals < 35 years of age, those who were >  = 75 years were at substantially higher risk of death with an OR2 (95% CI) of 10.13 (6.40–16.0). This association remained strong even after adjustment for baseline comorbidities, with OR3 (95% CI) of 7.43 (4.64–11.89). Other demographic predictors of mortality were male sex (OR2 = 1.48, 1.26–1.74) and EA background (OR2 = 1.35, 1.11–1.64) and patients in New York (OR2: 3.78, 2.94–4.86) compared to other geographic locations. Several baseline comorbidities were associated with increased risk of death, including presence of cardiac disease (OR = 1.55, 1.22–1.96), COPD (OR = 1.62, 1.25–2.08), or diabetes mellitus (OR = 1.59, 1.26–1.75), and use of immunosuppression (OR = 1.7, 1.12–2.79). Among presenting symptoms after adjusting demographics (OR2), comorbidities (OR3), only shortness of breath was associated with higher mortality, with OR (95% CI) of 1.91 (1.61–2.28). The presence of GI symptoms was not associated with mortality outcomes in this study. Several laboratory biomarkers were associated with increased risk of death, including elevated D-dimer (OR = 4.75, 2.82–8.02), elevated troponin (OR = 4.12, 3.26–5.22), lymphocytopenia (OR = 1.63, 1.32–2.00) or elevated lymphocytosis (OR = 2.53, 1.35–4.75), elevated CPK (OR = 1.60, 1.23–2.09), elevated CRP (OR = 4.00, 2.06–7.75), elevated procalcitonin (OR = 3.86, 3.04–4.90), elevated ferritin (OR = 2.32, 1.79–3.02), elevated LDH (OR = 3.36, 1.77–6.37), and elevated AST (OR = 2.63, 2.13–3.24). As for treatment interventions, glucocorticoid use was associated with a OR of 1.96 (1.61–2.39) for mortality from COVID-19 after adjusting for age/gender/race/center. In patients receiving mechanical ventilation, the OR for COVID-19 mortality was 15.44 (12.45–9.15) (Table 2).

## Discussion

The spectrum of COVID-19 disease ranges from asymptomatic to mild to severe, with the latter requiring hospitalization. In the early phases of the pandemic, severe COVID-19 disease often led to death. In this study, we collected data from 8 hospitals across the US with the goal of characterizing overall features and predictors of COVID-19 mortality among racially and ethnically diverse population. Our cohort consisted of 65% AA, 24.9% EA, 5% LAT, and 1.6% AS patients, with an overall 14.5% mortality. Among this cohort, we identified and verified predictors of death from COVID-19, including older age, male sex, comorbidities such as cardiac disease, COPD, diabetes mellitus, hypertension, and immune suppression, and respiratory symptoms like shortness of breath. Many of these factors such as older age [16], male sex [17], African American race [16, 18] and presence of comorbidities [16] have been previously identified in other studies for higher risk of mortality from COVID-19.

Although our cohort showed more females hospitalized, mortality was higher for males. This is consistent with other published data [6, 19, 20] and is likely due to gender differences in the level of expression of ACE2 and TMPRSS2 [17]. These two virulent factors are required by SARS-CoV-2 to gain entry into human cells for infection. EA had the highest mortality rate of 17% vs. 14.8% for AAs and 7.3% for LAT. This high death rate in EA (oldest group in our study) is further confirmed when OR for death were established for 6 age categories. The two age categories above 65 years maintained a high OR for death, even after multiple adjustments (Table 2). LAT were the youngest group in our study and had the lowest death rate of 7.3%. El Chaar et al.[21], reported increased death rates among AA and LAT in New York (Bronx—22.8% and Brooklyn—19.8%). These death rates are higher than our study results. This is probably secondary to the demographic variation of the locality, while ours consists of a large cohort that represents a national sample. The LAT patients in our study were the youngest and had the lowest mortality rate when compared to AA and EA. Greene et al. [22] reported decreasing age of SARS-CoV-2 positive patients in early versus later months of the pandemic, due primarily to increased testing and the fact that their study was on outpatients. When considering inpatients, as is the case in our study, we need to interpret the young age of the LAT as an indicator of higher exposure of this population, through living conditions and occupations in essential jobs that do not benefit from remote work protections that other populations have access to. This is further confirmed by the fact that EA in our cohort had the highest mortality which was associated with older age when compared to either one of the other racial groups. When adjusting for age, gender, and centers (OR2), EA had a higher OR of poor outcome when compared to AA, pointing to unknown genetic factors that might increase the risk of disease severity in this group.

Comorbidities also prove to be a risk factor for COVID-19 mortality. Primarily, a patient's susceptibility to SARS-CoV-2 may depend on a higher expression of ACE2, which has been found in patients with hypertension and cardiovascular disease [23]. Our study showed presence of pre-existing cardiovascular disease and hypertension was associated with poor out-comes in COVID-19 patients. Elevated plasma ACE2 activity is an independent predictor of major cardiac events associated with cardiovascular disease development, and ACE2 was found in carotid atherosclerosis and abdominal aortic aneurysm [24, 25]. Another mechanism that could affect the cardiovascular and other body systems during the COVID-19 pandemic is the acute systemic inflammatory response caused by the uncontrolled release of pro-inflammatory cytokines. The ACE2-related mechanism, Matrix-metalloproteinases control systems and cytokine levels are dysregulated in cardiovascular disease and COVID-19 [26]. This combined effect results in adverse clinical outcomes in COVID-19 patients with pre-existing cardiac diseases. Comorbid background allows progression of the disease to multi-system organ failure for eventual death of severe COVID-19 [27]. Comorbidities tend to be higher among AA populations, a set up for COVID-19 infection [16]. Here, these comorbidities also prove to be a risk factor for COVID-19 mortality. Comorbid background allows progression of the disease to multi-system organ failure for eventual death of severe COVID-19. Certain comorbidities such as asthma and diabetes mellitus lead to increased ACE2 and TMPRSS2 expression in lung cells [28]. Published data demonstrate that AA have twice the rate of hospitalization for Covid-19 compared to Whites [16]. However, once hospitalized (meaning severe COVID-19), there is no difference in death rates based on two large studies [29, 30]. Interestingly, in our cohort, we observed the highest mortality among AA and EA. EA patients were older in our cohort, which may have contributed to excess deaths in this group, as age is a strong predictor of death from COVID-19. After we adjusted for comorbidities, AAs had the highest risk of COVID-19 death. We did not find any correlation BMI and COVID-19 death.

In this study, Mortality rates were higher among patients in New York compared to other geographic locations. The crude fatality rate was 9.2% overall and 32.1% among hospitalized patients. New York was at the forefront of this pandemic and had limited resources at the time of data accrual, which could explain the differences in mortality rate. The high OR for COVID-19 death in New York relative to other locations also likely reflects existing health disparity.

There are mixed opinions regarding the usage of steroids in the treatment of COVID-19 patients. Glucocorticoid usage was associated with an increased risk of COVID-19 death in our cohort. This could be due to the inhibition of immune surveillance by corticosteroid leading to more persistent SARS-CoV-2 infection and higher viral load. While some studies have mentioned that glucocorticoids are used more routinely and are shown to be effective in treating severe COVID-19 patients and showed a reduction in the need for mechanical ventilation since the beginning of the pandemic [31, 32], other studies mentioned that corticosteroid use in critically ill COVID-19 patients was associated with a much higher case fatality rate [33, 34]. Glucocorticoid treatment delayed viral clearance and increase in secondary infections in COVID-19 patients of taking high doses or medium doses instead of low doses of glucocorticoids [31, 33]. We recommend judicious use of glucocorticoids in mild to moderate COVID-19 patients.

One of the unique aspects of our study is the inclusion of serum biomarkers with comprehensive symptomatic, clinical, as well as racial and ethnic information. Anemia, as well as lymphopenia and lymphocytosis reflect bone marrow effects due to acute viral infection. Anemia can exacerbate hypoxia among COVID-19 patients [35]. COVID-19 is a systemic inflammatory disease, and we identified several inflammatory markers and acute phase reactants as risk, including C-reactive protein, procalcitonin, and ferritin. COVID-19 creates a prothrombotic milieu or results in a prothrombotic, and consistent with this, we noted high risk for death with elevation of D-dimers. SARS-CoV-2 can infect other organs besides the lung, as well as can cause secondary injury to organs as a result of respiratory failure [35–38]. We noted elevated risk of death from COVID-19 when patients had elevated troponins, indicating heart muscle damage. Heart muscle, as well as skeletal muscle, can release CPK and AST, and elevation of both individually was associated with increased risk of death. Liver inflammation can occur with COVID-19, but may be a bystander effect that is not necessarily a contributor towards COVID-19 mortality [39]. In our cohort, we did not observe a risk of death with elevation of ALT, which is more specific to the liver. Elevation of LDH, a marker of tissue damage from metabolic dysfunction, was also associated with risk of COVID-19 death.

We note some differences in prevalence and frequency of clinical presentation of severe COVID-19 among hospitalized patients. AAs had the highest prevalence of comorbidities and inflammatory markers, and LAT patients had the highest prevalence of symptoms. It is unclear why there might be differences in presentation, particularly for symptoms, between certain races/ethnicities. While there is evidence that comorbidities may foster increased ACE2 and TMPRSS2 expression leading to higher chance for SARS-CoV-2 infection loads [28], it does not necessarily explain symptomology differences. We do not know if there is biology behind the differences or cultural.

This study has some limitations. That is retrospective and the fact that it involves areas in the US that were hit by the pandemic at different times. This might have affected our findings as the New York area that was hit first and hard by the pandemic displayed higher mortalities than other regions that might have had more time understanding and better managing COVID-19 patients for better outcome. There was a lack in some laboratory parameters such as LDH, CPK, and procalcitonin from all the centers. So, the analysis of these factors associated with outcomes probably suffered from confusion bias. However, the analyses were consistent with what was known elsewhere, and this external consistency has the benefit of increasing confidence that the data are robust. It is therefore believed that the main results, which are the overall and group mortality rates are plausible.

## Conclusions

Our study encompasses symptomatic, clinical, and biomarker associations among races/ethnicities for COVID-19 death. Our cohort was enriched for AA patients by virtue of the 8 hospitals that could provide information for this study. We identify and verify several aspects for risk of COVID-19 death, and enhance, and enrich the literature with simultaneous associations from serum biomarkers that can add to improve patient care for prediction of death from COVID-19. We recommend special attention is required in treating COVID-19 African American Population. Priority should be given to patients with elevated inflammatory markers, preexisting conditions and elevated liver enzymes. We also recommend judicious use of glucocorticoids in mild to moderate COVID-19 hospitalized patients.

## Data Availability

All data generated or analyzed during this study are included in this published article.
