# Correction: Symptomatic, clinical and biomarker associations for mortality in hospitalized COVID-19 patients enriched for African Americans

**DOI:** 10.1186/s12879-022-07699-3

**Published:** 2022-08-29

**Authors:** Hassan Ashktorab, Antonio Pizuorno, Folake Adeleye, Adeyinka Laiyemo, Maryam Mehdipour Dalivand, Farshad Aduli, Zaki A. Sherif, Gholamreza Oskrochi, Kibreab Angesom, Philip Oppong-Twene, Suryanarayana Reddy Challa, Nnaemeka Okorie, Esther S. Moon, Edward Romos, Boubini Jones-Wonni, Abdoul Madjid Kone, Sheldon Rankine, Camelita Thrift, Derek Scholes, Chiamaka Ekwunazu, Abigail Banson, Brianna Mitchell, Guttu Maskalo, Jillian Ross, Julencia Curtis, Rachel Kim, Chandler Gilliard, Geeta Ahuja, Joseph Mathew, Warren Gavin, Areeba Kara, Manuel Hache-Marliere, Leonidas Palaiodimos, Vishnu R. Mani, Aleksandr Kalabin, Vijay Reddy Gayam, Pavani Reddy Garlapati, Joseph Miller, Lakshmi Gayathri Chirumamilla, Fatimah Jackson, John M. Carethers, Farin Kamangar, Hassan Brim

**Affiliations:** 1 Department of Medicine, GI Division, Cancer Center, Howard University Hospital, NW, 2041 Georgia Avenue, DC Washington, USA; 2 Department of Pathology and Cancer Center, Department of Biochemistry and Molecular Biology, Howard University College of Medicine, Washington, DC USA; 3 College of Engineering and Technology, American University of the Middle East, Salmiya, Kuwait; 4 Division of General Internal Medicine and Geriatrics, Indiana University School of Medicine, Indianapolis, IN USA; 5 Department of Medicine, Albert Einstein College of Medicine, Bronx, NY USA; 6 Department of Trauma, Acute and Critical Care Surgery, Duke University Medical Center, Durham, NC USA; 7 Department of Surgery, Columbia University College of Physicians and Surgeons at Harlem Hospital, New York, NY USA; 8 Department of Medicine, Interfaith Medical Center, New York, NY USA; 9 Departments of Emergency Medicine and Internal Medicine, Henry Ford Hospital, Detroit, MI USA; 10 Division of Gastroenterology and Hepatology, Department of Internal Medicine, Department of Human Genetics and Rogel Cancer Center, University of Michigan, Ann Arbor, MI USA; 11 Department of Biology, School of Computer, Mathematical, and Natural Sciences, Morgan State University, Baltimore, MD USA

## Correction to: BMC Infectious Diseases (2022) 22:552 https://doi.org/10.1186/s12879-022-07520-1

Following publication of the original article [1], the authors identified an error in the author Geeta Ahuja.

The incorrect author name is: Geetha Ahuja.

The correct author name is: Geeta Ahuja.

The author group has been updated above and the original article [1] has been corrected.
